# MRI predictors for the conversion from contrast-enhancing to iron rim multiple sclerosis lesions

**DOI:** 10.1007/s00415-022-11082-2

**Published:** 2022-03-25

**Authors:** Nicolas Wenzel, Matthias Wittayer, Claudia E. Weber, Lucas Schirmer, Michael Platten, Achim Gass, Philipp Eisele

**Affiliations:** 1 Department of Neurology, Medical Faculty Mannheim and Mannheim Center of Translational Neurosciences (MCTN), Heidelberg University, Theodor-Kutzer-Ufer 1-3, 68167 Mannheim, Germany; 2 Institute for Innate Immunoscience, Medical Faculty Mannheim, Heidelberg University, Mannheim, Germany; 3 DKTK CCU Neuroimmunology and Brain Tumor Immunology, DKFZ, Heidelberg, Germany

**Keywords:** Multiple sclerosis, Magnetic resonance imaging, Iron rim lesion, Chronic active lesions

## Abstract

**Background:**

In multiple sclerosis (MS), iron rim lesions (IRLs) are characterized by progressive tissue matrix damage. Therefore, early identification could represent an interesting target for therapeutic intervention to minimize evolving tissue damage. The aim of this study was to identify magnetic resonance imaging (MRI) parameters predicting the conversion from contrast-enhancing to IRLs.

**Methods:**

We retrospective identified MS patients scanned on the same 3 T MRI system presenting at least one supratentorial contrast-enhancing lesion (CEL) and a second MRI including susceptibility-weighted images after at least 3 months. On baseline MRI, pattern of contrast-enhancement was categorized as “nodular” or “ring-like”, apparent diffusion coefficient (ADC) maps were assessed for the presence of a peripheral hypointense rim. Lesion localization, quantitative volumes (ADC, lesion volume) and the presence of a central vein were assessed.

**Results:**

Eighty-nine acute contrast-enhancing lesions in 54 MS patients were included. On follow-up, 16/89 (18%) initially CELs converted into IRLs. CELs that converted into IRLs were larger and demonstrated significantly more often a ring-like contrast-enhancement pattern and a peripheral hypointense rim on ADC maps. Logistic regression model including the covariables pattern of contrast-enhancement and presence of a hypointense rim on ADC maps showed the best predictive performance (area under the curve = 0.932).

**Discussion:**

The combination of a ring-like contrast-enhancement pattern and a peripheral hypointense rim on ADC maps has the ability to predict the evolution from acute to IRLs. This could be of prognostic value and become a target for early therapeutic intervention to minimize the associated tissue damage.

## Introduction

In multiple sclerosis (MS), acute contrast-enhancing lesions (CELs) on T1-weighted post-contrast magnetic resonance imaging (MRI) represent characteristic hallmarks and are considered markers of blood–brain barrier breakdown, often facilitating the fulfillment of dissemination in time in the diagnosis of MS [1] or demonstrating ongoing disease activity despite immune treatment, but they do not correlate with the development of cumulative impairment or disability [2].

There has been an increasing interest in “chronic active” or “smoldering” lesions, indicating ongoing disease activity in the absence of contrast-enhancement [3]. Chronic active lesions are characterized by progressive central nervous system matrix damage, driven by a proinflammatory rim of iron-laden microglia/macrophages and reactive astrocytes at the lesion edge [4, 5]. In vivo, chronic active lesions can be visualized as “paramagnetic rim lesions” or “iron rim lesions” (IRLs) on susceptibility-weighted imaging (SWI) [5–7]**.** IRLs are associated with higher disease severity [7, 8], brain [7, 8] and spinal cord atrophy [8] and have been suggested as new imaging biomarker of disease progression. Therefore, early identification of these lesions could represent a therapeutic target to minimize associated neuronal tissue damage. The aim of this study was to identify conventional and advanced MRI parameters predicting the conversion from acute contrast-enhancing to iron rim MS lesions.

## Methods

### Patients

We retrospectively screened our database to identify patients fulfilling the following inclusion criteria: (1) diagnosis of definite MS according to the 2010 diagnostic criteria [9]; (2) at least 18 years of age; (3) a 3 T dataset acquired on the same MRI system including a 3D magnetization-prepared rapid acquisition gradient-echo (MPRAGE) sequence, a 3D fluid-attenuated inversion recovery (FLAIR)-data set, diffusion-weighted images including apparent diffusion coefficient (ADC) calculations and post-contrast T1-weighted images; (4) presence of at least one supratentorial CEL and (5) a second MRI (follow-up MRI) including SWI acquired on the same MRI system after at least 3 months. Trained neurologists assessed MS patients using the Expanded Disability Status Scale (EDSS) score on the days of the MRI examinations.

### Magnetic resonance imaging

MRI was performed on a 3 T MR system (MAGNETOM Skyra, Siemens Healthineers, 20-channel head coil) including the following sequences: 3D MPRAGE (echo time (TE) = 2.49 ms, repetition time (TR) = 1900 ms, inversion time (TI) = 900 ms, field-of-view (FOV) = 240 mm, spatial resolution = 0.9 × 0.9 × 0.9 mm), 3D FLAIR (TE = 398 ms, TR = 5000 ms, TI = 1800 ms, FOV = 240 mm, resolution = 0.5 × 0.5 × 0.9 mm), diffusion-weighted echo planar images (TE = 68 ms, TR = 5300 ms, b = 0/1000 s/mm^2^, FOV 220 mm, slice thickness 4 mm, resolution = 0.98 × 0.98 × 4.0 mm) including ADC calculations and T1-weighted images (TE = 2.5 ms, TR = 225 ms, FOV = 220 mm, slice thickness = 3 mm, voxel size 0.7 × 0.7 × 3.0 mm) acquired 10 min after contrast-injection (single dose gadoterate meglumine). Until September 2018, SWI (TE = 20 ms, TR = 27 ms, FOV = 220 mm, slice thickness (ST) = 1.5 mm, voxel-size 0.9 × 0.9 × 1.5 mm) were acquired in our department after contrast-injection as a “delay” before acquisition of post-contrast T1-weighted images, afterwards prior contrast agent administration.

### Post-processing analysis

**Fig. 1:**
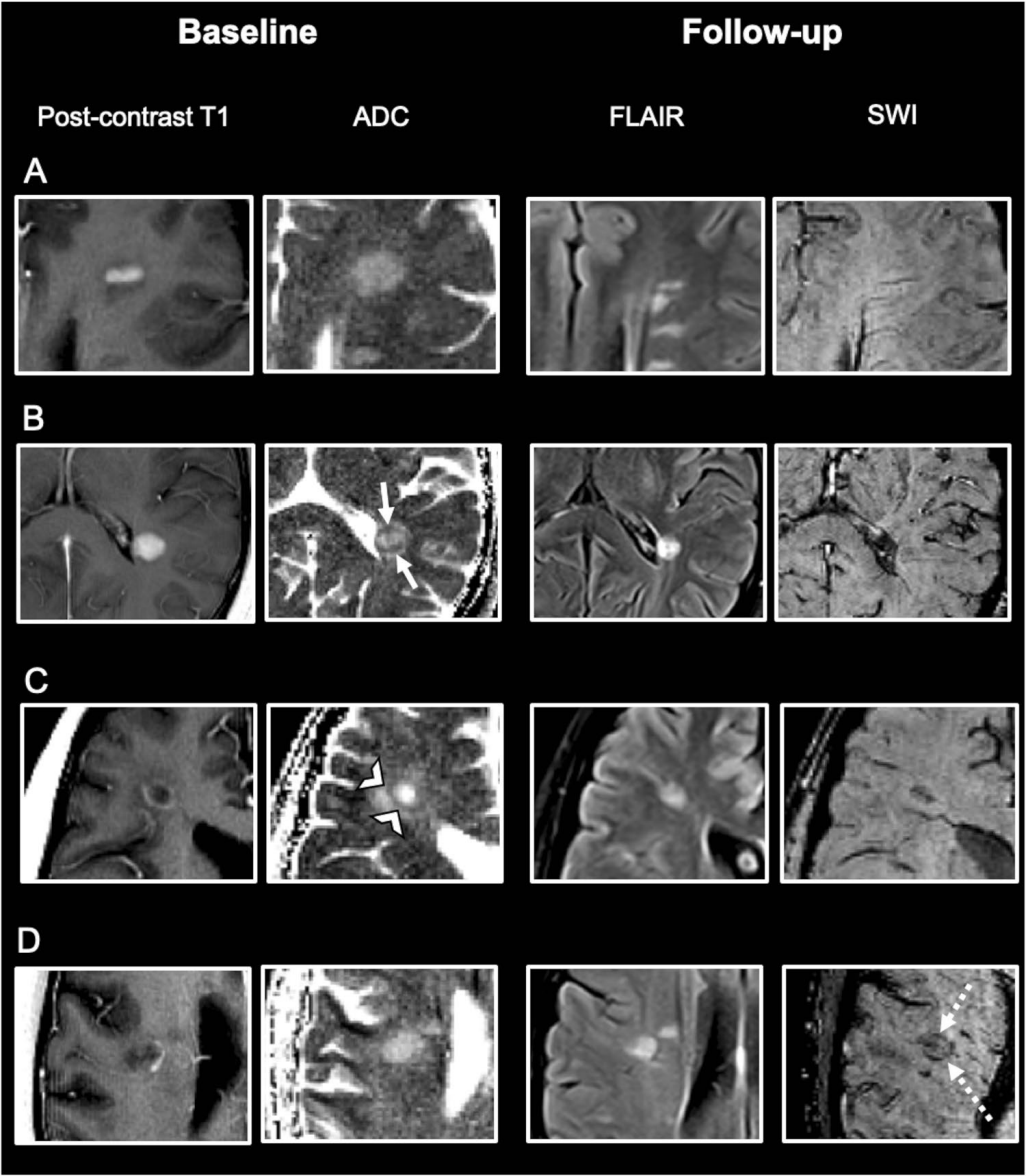
Representative examples of the observed contrast-enhancement and apparent diffusion coefficient (ADC) patterns of acute multiple sclerosis lesions. From left to right: Baseline post-contrast T1-weighted images and baseline ADC maps. Fluid-attenuated inversion recovery (FLAIR) and susceptibility-weighted images (SWI) at follow-up. **A** Nodular enhancement without a hypointense rim on ADC maps. On follow-up MRI after 9 months, no iron rim is detectable. **B** Nodular contrast-enhancement with a hypointense rim on ADC maps (arrows). On follow-up MRI after 3 months, no iron rim is detectable. **C** Ring-like enhancement with a hypointense rim on ADC maps (open arrowheads). On follow-up MRI after 6 months, no iron rim is detectable. **D** Ring-like enhancement without a hypointense rim on ADC maps. Note the iron rim on follow-up after 14 months (dotted arrows)

In all patients, MR images were evaluated in consensus by two readers (with 2 and 15 years’ experience respectively). If uncertainty remained regarding potential lesion classification, an additional reviewer (with 30 years’ experience) was consulted for final determination of lesion classification. On baseline MRI, acute CELs were identified on post-contrast T1-weighted images. To minimize partial volume effects only supratentorial lesions ≥ 5 mm in their longitudinal axis entered further analysis. According to their topography, lesions were classified as periventricular, deep white matter or juxtacortical (located in the frontal, parietal, temporal or occipital lobe) [10]. Pattern of contrast-enhancement was categorized as “nodular” or “ring-like” as suggested previously [11–13]. ADC maps were assessed for the presence of a peripheral hypointense rim [14]. Figure 1 demonstrates representative examples. The initially CELs were identified on follow-up MRI and investigated for the presence of a hypointense rim on SWI [15]. In cases hypointense ring-like signals or “dots” at the lesion edge were visible on at least two contiguous slices, the initially CEL was defined as an IRL, otherwise as a non-IRL. In addition, SWI minimum intensity projection images were carefully evaluated to guarantee that venous vessels at the lesion edge were not mistaken for part of iron rims. Furthermore, susceptibility-weighted images were evaluated for the presence of the central vein sign (CVS) according to the “North American Imaging in Multiple Sclerosis Cooperative” (NAIMS) criteria [16].

Post-contrast T1-weighted images and ADC maps were co-registered onto the 3D MPRAGE images [17, 18]. CELs were outlined with a semi-automated assistance using the drawing tool of MRIcroGL (https://nitrc.org/projects/mricrogl). Lesion outlines were applied to the co-registered quantitative ADC maps. Mean quantitative parameters (lesion volumes, ADC values) were extracted for each lesion and used for statistical assessment.

To determine baseline brain volumes, normalized for subject head size, 3D MPRAGE data sets were applied to the automated model-based segmentation tool, SIENAX [19, 20], part of FSL [21]. To correct for misclassification of T1-weighted grey matter volume in the presence of high T1-weighted hypointense lesion volume, T1-weighted hypointense lesions were filled with the mean intensity value of the normal appearing white matter present in the same slice of the lesion. Calculation of deep grey matter (DGM) volumes was performed with FSL FIRST [22]. Total T2-hyperintense and T1-hypointense lesion volumes (on 3D FLAIR and 3D MPRAGE datasets respectively) were quantified using the drawing tool of MRIcroGL.

### Statistical analysis

To assess variables predicting the evolution of IRLs, we calculated a logistic model with all variables acquired using the forward inclusion algorithm, using the likelihood-quotient as goodness of fit criterion (SPSS, Version 28, SPSS Inc., Chicago, IL, USA).

### Standard protocol approvals, registrations and patient consents

This study was approved by the local ethics committee (Ethikkommission II, Medical Faculty Mannheim, Heidelberg University, 2017-830R-MA) and performed in accordance with the ethical standards laid down in the 1964 Declaration of Helsinki and its later amendments. Patient consent was waived due to the retrospective nature of the study and the lack of patient interaction.

## Results

Baseline characteristics of the study population and radiological data are presented in Table 1. A total of 54 MS patients (all relapsing–remitting) were included in the final analysis. On baseline MRI, 22/54 (41%) patients demonstrated at least one IRL (mean 0.8 IRLs per patient, range 0–7). Overall, we identified 89 acute CELs on post-contrast T1-weighted MRI (range 1–7 per patient). Twenty-nine (33%) lesions were classified as periventricular, 48 (54%) as deep white matter and 12 (13%) as juxtacortical lesions. Seventy-one lesions (80%) showed a nodular, the remaining 18 (20%) a ring-like contrast-enhancement pattern. A peripheral hypointense rim on ADC maps was observed in 21/89 (24%), a CVS in 57/89 (64%) lesions. In 12/18 (67%) ring-like enhancing lesions, contrast-enhancement co-localized with the hypointense rim on ADC maps. Follow-up MRI was performed after a mean of 13.31 ± 13.12 months (range 3–59 months), in 45/54 patients, SWI were acquired after contrast-injection.

**Table 1:** Baseline characteristics of the study population

Number of patients	54
Gender, *n* (female/male)	37/17
Age, years, mean ± SD	32.28 ± 10.2
EDSS, median (range)	2.0 (0–7.5)
Disease duration, years, mean ± SD	3.72 ± 4.76
Relapse at time point of MRI, *n* (%)	24 (44%)
Disease modifying therapy, *n* (%)	37 (69%)
Grey matter volume (mL), mean ± SD	773.75 ± 72.92
White matter volume (mL), mean ± SD	751.34 ± 65.99
Deep grey matter volume (mL), mean ± SD	34.44 ± 4.43
T2 lesion volume (mL), mean ± SD	6.76 ± 6.5
T1 lesion volume (mL), mean ± SD	4.03 ± 4.89
Time to follow-up, months, mean ± SD (range)	13.31 ± 13.12 (3 – 59)

*EDSS* Expanded Disability Status Scale, *MRI* magnetic resonance imaging, *SD* standard deviation

On follow-up, 16/89 (18%) initially CELs converted into IRLs. CELs that converted into IRLs demonstrated significantly more often a ring-like contrast-enhancement pattern (13/16 (81%) versus 5/73 (7%); *p* < 0.001), a peripheral hypointense rim on ADC maps (12/16 (75%) versus 9/73 (12%); *p* < 0.001) and a CVS (15/16 (94%) versus 42/73 (58%); *p* < 0.001) compared to acute lesions that converted into non-IRLs. Furthermore, CELs converting into IRLs were significantly larger (1.17 ± 1.13 mL versus 0.47 ± 0.52 mL; *p* < 0.001), whereas ADC values (0.99 ± 0.09 × 10^–3^ mm^2^/s versus 0.96 ± 0.16 × 10^–3^ mm^2^/s) and lesion localization did not differ between the two groups (*p* > 0.05 for both comparisons). None of the initially CELs demonstrated a peripheral hypointense rim on ADC maps at follow-up. On follow-up MRI, in 24/54 (44%) patients at least one IRL was detectable (mean 1.1 IRL per patient; range 0–8).

The final logistic regression model that was evolved by the forward inclusion procedure showed significant improvement of the likelihood quotients only when including the pattern of contrast-enhancement and the presence or absence of a hypointense rim on ADC maps. The model was significant (*χ*^2^ 44.403, *p* < 0.001) and showed a large amount of explained variance (Nagelkerkes *R*^2^ 0.644).

**Fig. 2:**
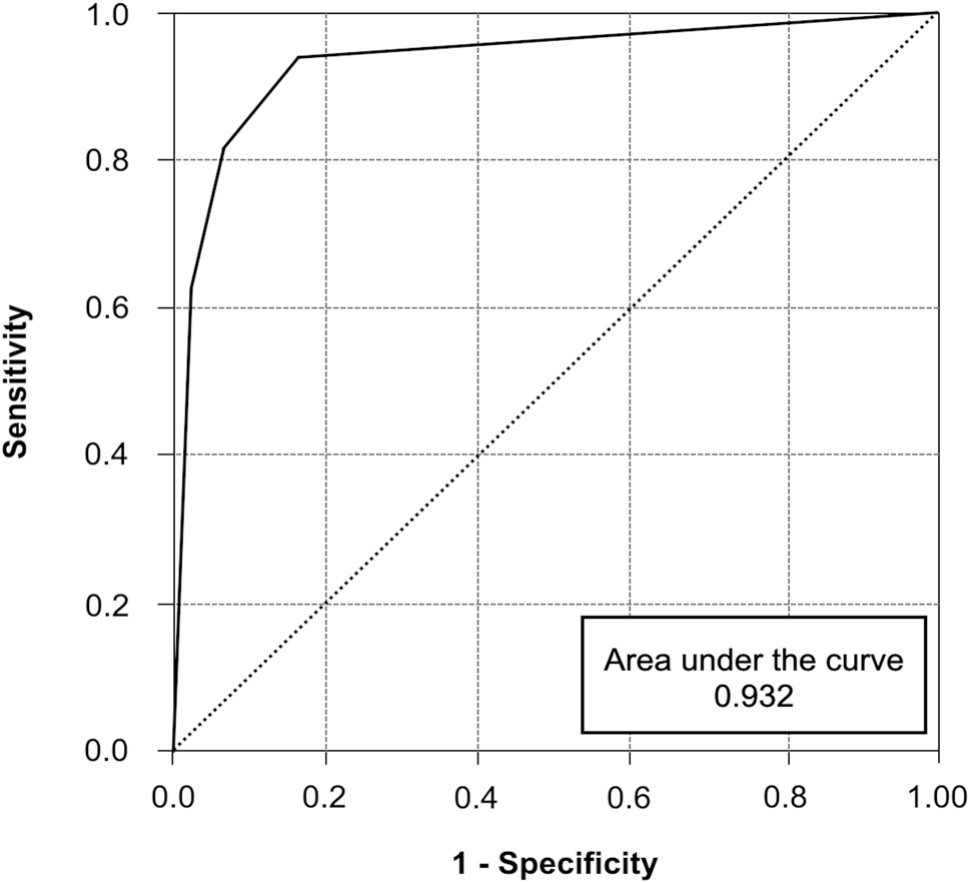
Receiver-operating-characteristics (ROC) of the logistic regression model including the covariables pattern of contrast-enhancement and presence of a hypointense rim on apparent diffusion coefficient maps

**Fig. 3:**
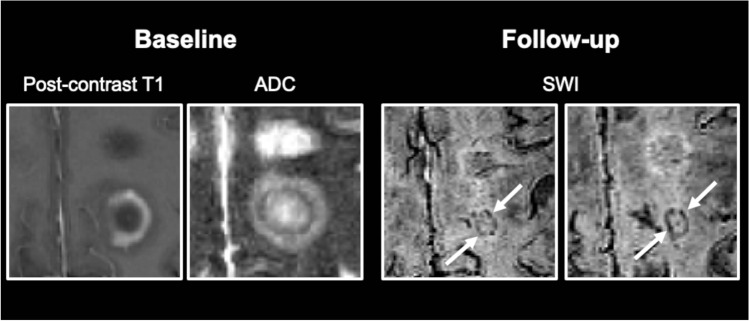
Representative example of an acute multiple sclerosis lesion presenting with a ring-like contrast-enhancement pattern, accompanied by a hypointense rim on maps of the apparent diffusion coefficient (ADC) that converts into an iron rim lesion on susceptibility-weighted images (SWI) during follow-up (arrows)

We included the probabilities from the model estimation in a receiver operating characteristics (ROC) analysis, which showed a good predictive performance with an area under the curve (AUC) = 0.932; see Fig. 2. Figure 3 demonstrates a representative example of an acute lesion with a ring-like enhancement-pattern and a peripheral hypointense rim on ADC maps that converted into an IRL on follow-up.

## Discussion

In this study, we investigated potential MRI parameters in MS predicting the conversion of acute contrast-enhancing into chronic active IRLs. Our results demonstrate that the combination of a ring-like contrast-enhancement pattern and a peripheral hypointense rim on ADC maps shows an excellent accuracy (AUC of 0.932) to predict the evolution from acute to IRLs.

Diffusion-weighted MRI represents a sensitive quantitative marker of microstructural tissue damage. Several studies have reported the characteristics of acute demyelinating MS lesions presenting with reduced diffusion [14, 23–28]. Besides a temporal evolution of diffusion signal changes [23, 28], acute MS lesions may show spatial signal variations with reduced diffusion in the periphery and increased diffusion in the lesion core [14]. A reduction of water molecule diffusion accompanied on MRI with hyperintense DWI signal and reduced ADC values is a result of mitochondrial dysfunction causing disturbance of energy metabolism due to cytotoxic cell swelling because of an aggressive inflammatory milieu, induced by proinflammatory cytokines including complement factors such as C3 and C1qA, TNF-alpha and nitric oxide [29–31]. Reduced diffusion in the periphery of acute MS lesions may be furthermore due to the presence of cytotoxic edema of oligodendroglia and/or dense cellular infiltrates consisting of iron-laden macrophages at the lesion edge, resulting in a reduced extracellular space [14]. The pathological hallmark of IRLs is the ongoing tissue destruction driven by peripheral blood mononuclear cells including proinflammatory microglia and macrophages assumed to block remyelinating mechanisms [5–7, 32]. Previous studies demonstrated that these macrophages and microglia at the lesion edge are characterized by enhanced antigen presentation, myelin phagocytosis and expression of activation markers such as CD163, MSR1, CD68 [6, 7, 33] and p22phox [5]. Furthermore, CD68-positive demyelinating inflammatory infiltrates distribute along veins crossing the lesion edge [6]. Using MRI-informed single-cell RNA sequencing, a recent study provided a comprehensive map of immune and glial cells involved in IRLs, as well as their corresponding gene expressions and potential interactions [34]. In particular, the authors demonstrated that C1q was mainly expressed by a subgroup of microglia responsible for driving inflammation [34]. Therefore, one could hypothesize that a potentially neurotoxic inflammatory milieu at the time point of contrast-enhancement (as demonstrated by a hypointense rim on ADC maps), triggers a cascade to a self-sustained low degree of chronic inflammation in IRLs. Interestingly, in a recent experimental study, blocking C1q reduced iron-containing microglia in mice suggesting that C1q inhibition represents a potential therapeutic target to address chronic inflammation [34].

In our study, a hypointense lesion rim on ADC maps was detectable in 21/89 lesions (24%), a finding that is comparable to previous findings [14], whereas we observed this pattern significantly more often in contrast-enhancing that transitioned into IRLs during follow up. Interestingly, a previous study demonstrated that peripheral reduced diffusion in acute demyelinating lesions is frequently observed in ring-enhancing lesions on post-contrast MRI [14]. Of note, in our study, not all IRLs with an initial hypointense rim on ADC maps also demonstrated a ring-like contrast-enhancement pattern (and vice versa), suggesting that both MRI patterns do not represent an epiphenomenon but rather two distinct underlying pathologies. Therefore, combining the information provided by diffusion-weighted and contrast-enhanced MRI might provide new insights into IRL characteristics.

According to their pattern, CELs have been traditionally classified as “nodular” or “ring-like” [11–13] and previous studies suggested that ring-like lesions are associated with severe neuronal tissue loss [11, 12]. Our results would be in line with these observations since acute lesions demonstrating a ring-like enhancement pattern became more often IRLs during follow up, which in turn are associated with pronounced tissue damage [5–7]. In contrast to static MRI, dynamic contrast-enhanced (DCE) MRI has the ability to demonstrate spatiotemporal dynamics of contrast-enhancement within lesions, reflecting blood–brain barrier opening [35]. Using DCE MRI, previous studies demonstrated that initially ring-like lesions enhance centripetally and later fill in (in some cases only partially) and initially nodular lesions enhance centrifugally [35–37]. Interestingly, lesions demonstrating an initial nodular enhancement were smaller than lesions with an initial ring-like enhancement [36, 37], a finding that is consistent with the results of our study. Furthermore, a previous study showed that centripetal DCE lesions are accompanied by a rim at the lesion edge, whereas no rim was observed in centrifugally enhancing lesions [6]. However, due to the retrospective design of our study, no DCE MRI sequences were available. Therefore, we can only hypothesize that ring-like lesions in our study cohort would enhance centripetally, whereas nodular lesions would enhance centrifugally.

Several limitations also need to be mentioned: the rim thickness in chronic active MS lesions at 7 T MRI has been estimated at ~ 430 µm [35] and the in-plane voxel dimensions of the axial SWI used in our study (0.9 × 0.9 × 1.5 mm) were larger compared to previous studies [6, 7]. At ultra-high field 7 T, potentially more susceptibility effects (iron rims, veins) might be detectable. However, a previous study demonstrated that nearly all 7 T paramagnetic rims can also be found at 3 T [38]. Furthermore, the spatial resolution of the axial DWI was lower than the resolution of the other sequences, which theoretically could introduce partial volume effects. Only 16/89 (18%) CELs converted into IRLs during follow-up, a finding that is in line with a previous study [6]. Due to the small sample size, interpretation of our results should be done cautiously. A previous study demonstrated that in some lesions the iron rim disappears within the first 3 months after contrast-enhancement [6]. Therefore, one of our inclusion criteria contained a follow-up MRI including SWI after at least 3 months. In addition, more recent studies showed that in a small percentage of IRLs the rim even wanes and finally disappears during long-term follow-up [39, 40]. Future studies including more CELs and longer follow-up observation time points are recommended.

Our results suggest that the combination of a ring-like contrast-enhancement pattern and a lesion-surrounding hypointense rim on ADC maps predicts the evolution from acute CELs to chronic active lesions in MS. Early identification of those lesions could be of prognostic value and could represent an interesting target for early therapeutic intervention to minimize the associated tissue damage.
